# Experimental Studies on MoS_2_-Treated Grinding Wheel Active Surface Condition after High-Efficiency Internal Cylindrical Grinding Process of INCONEL^®^ Alloy 718

**DOI:** 10.3390/mi10040255

**Published:** 2019-04-17

**Authors:** Wojciech Kapłonek, Krzysztof Nadolny, Marzena Sutowska, Mozammel Mia, Danil Yurievich Pimenov, Munish Kumar Gupta

**Affiliations:** 1 Department of Production Engineering, Faculty of Mechanical Engineering, Koszalin University of Technology, Racławicka 15-17, 75-620 Koszalin, Poland; wojciech.kaplonek@tu.koszalin.pl (W.K.); marzena.sutowska@tu.koszalin.pl (M.S.); 2 Department of Mechanical and Production Engineering, Ahsanullah University of Science and Technology, 141-142, Love Road, Tejgaon I/A, Dhaka 1208, Bangladesh; mozammel.mpe@aust.edu; 3 Department of Automated Mechanical Engineering, South Ural State University, Lenin Prosp. 76, 454080 Chelyabinsk, Russia; danil_u@rambler.ru; 4 University Center for Research & Development, Department of Mechanical Engineering, Chandigarh University, Gharuan, Punjab 140413, India; munishguptnit@gmail.com

**Keywords:** impregnation process, molybdenum disulfide, abrasive tools, hard-to-cut materials, measurement systems, image processing and analysis

## Abstract

This work demonstrates that molybdenum disulfide can be successfully used as an impregnating substance that is introduced in the abrasive tool structure for improving its cutting properties and favorably affecting the effects of the abrasive process. For the experimental studies, a set of MoS_2_-treated small-sized grinding wheels with a technical designation 1-35×10×10×109A5X60L10VE0 PI-50 before and after the reciprocating internal cylindrical grinding process of rings made from INCONEL^®^ alloy 718 was prepared. The condition of grinding wheel active surface was analyzed using an advanced observation measurement system based on stylus/optical profilometry, as well as confocal and electron microscopy. The obtained results confirmed the correctness of introduction of the impregnating substance into the grinding wheel structure, and it was possible to obtain an abrasive tool with a given characteristic.

## 1. Introduction

The introduction of advanced high-efficiency abrasive technologies into the modern machine, automotive, and aeronautic industries, as widely described by Hou et al. and Wang and Li [1,2], forced the producers of abrasive tools to constantly improve them, as well as to search for more effective abrasive materials, allowing an increase of the efficiency and stability of the machining process and extending the tool life. One of the key factors with a decisive influence on the stable course of the grinding process [3] (and hence its efficiency) is the temperature increase in the grinding zone (GZ). In the case of grinding wheels, it was described in detail by Xu et al., as well as by Malkin and Guo [4,5], whereas it was described for multilayer metal composite structures by Pashnyov et al. [6]. Another important factor is the cutting force (CF). Pereverzev and Pimenov [7] showed the impact of the conditions of grinding and flank wear of the grinding wheel on the CF. In order to minimize the impact of temperature and the resulting intensification of many unfavorable phenomena, which may strongly interfere with the machining process and make it difficult to get the intended effects of machining, solid lubricant substances are deliberately introduced into the GZ, e.g., solid grease Kool-Grid G-440 (Grangerg Int., Pittsburg, CA, USA) or antiadhesive substances. This introduction can be realized by using a specially constructed tool equipped with inserts of solid grease, allowing the deposition of a solid lubricant substance on the grinding wheel active surface (GWAS) during the abrasive process or a substance in the form of a suspension with cooling liquid (CL) through special nozzles, as reported by Shaji and Radhakrishnan [8,9]. Another way is to introduce lubricant substances in the form of fillers as integral components of an abrasive tool, not involving the use of abrasive grains or a binder, as presented by Shaji and Radhakrishnan, as well as Tsai and Jian [8,10]. The above modification is most often used during the production of grinding wheels with a resin binder, because the curing temperature of the binder is relatively low (approximately 200–250 °C (392–482 °F)), resulting in thermal decomposition of the filler. In the case of grinding wheels with a ceramic binder, such a treatment is very limited due to the high sintering temperature (approximately 1200 °C (2192 °F), which creates a high risk of the decomposition of lubricant substances and/or antiadhesives during the process. In such situations, the impregnation of grinding wheels is used.

**Figure 1:**
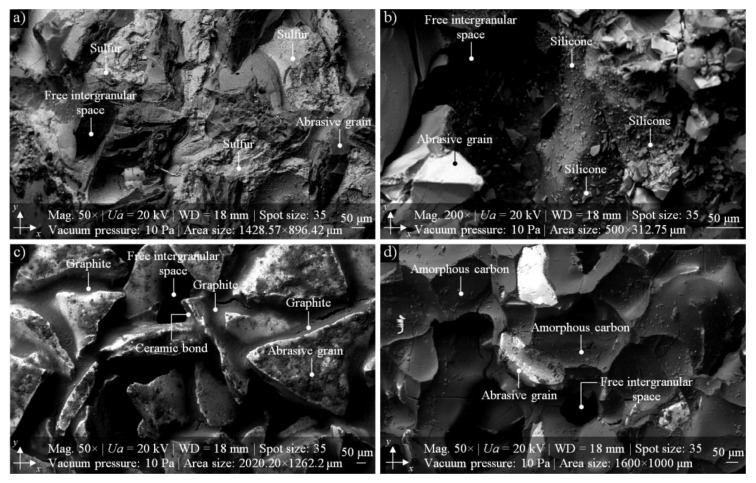
Collection of SEM micrographs obtained using a scanning electron microscope JEOL JSL-5500LV, presenting examples of the grinding wheel active surface (GWAS) impregnated with popular impregnating substances: (**a**) sulfur; (**b**) silicone; (**c**) graphite; (**d**) amorphous carbon [43,44].

The process of impregnation of abrasive tools was established over 90 years ago and it is successfully used in many branches of the modern machine industry, related to, e.g., high-grade steel processing, rolling bearing production, and non-ferrous metal machining. The impregnation process, described in detail by Marinescu et al., Malkin and Guo, and Dresel and Mang [11,12,13], is realized through the use of various types non-toxic substances based on the introduction of non-metallic elements (sulfur, allotropic varieties of carbon) and metals (copper, bismuth), synthetic organosilicon polymers (silicone), natural resins (rosin), and synthetic resins (epoxy resin), as well as others substances (paraffin, wax), into the GWAS. Of the many substances listed above, the most important are presented in Table 1, whereas SEM micrographs of selected ones are presented in Figure 1.

The world’s leading manufacturers of abrasive tools (e.g., Atlantic, Hermes Schleifmittel, Joto Abrasives, Norton, Sercap) are eager to take advantage of the possibility of commercially producing impregnated abrasive tools and bringing them onto the market. They are available on offer in the current catalog, and they can also be produced for individual customer orders.

One of the simplest and most widely used industrial practice methods of impregnation of abrasive tools involves the direct introduction of a molten impregnation substance into their active surface. After the impregnation cools, the grinding wheel can then be used immediately to carry out the abrasive process.

The most popular grinding wheels impregnated with sulfur or wax have the following major disadvantages:

- The impregnating substance fills all intergranular free spaces, which reduces the ability to transport the CL to the GZ and to remove the products of the process;
- The apparatus used for carrying out the impregnation of abrasive wheels is complicated and creates many problems during its operation.

In previous works of Nadolny et al. [22], Nadolny et al. [43], and Kapłonek et al. [44], a method was suggested that solved the first problem. It consisted of a partial centrifugation of the liquid impregnating substance, which prevented its introduction into intergranular free spaces. This solution, however, caused problems related to the centrifugation process, including the need to constantly control the temperature during its course, which was dictated by the real possibility of an autoignition of the impregnating substance (sulfur, paraffin). These problems prompted the authors to develop a different, more universal impregnation method for ceramic abrasive tools, potentially applicable by not only tool manufacturers, but also their users, who could adapt the impregnate composition to current technological needs.

This work presents the above method used for the molybdenum disulfide impregnation process of 1-35×10×10×109A5X60L10VE01PI-50 grinding wheels (Section 2.2). The MoS_2_-treated grinding wheels were tested with the reciprocal peripheral internal cylindrical grinding process of INCONEL^®^ alloy 718 (Section 2.3). The results of these experiments were evaluated by a detailed collection of measurements and analyses carried out through the use of advanced contact and non-contact measurement systems (Section 2.4). In the final part of the work, a summary of all obtained results with their detailed interpretation (Section 3.1, Section 3.2 and Section 3.3) is presented.

## 2. Materials and Methods

### 2.1. Main Goal

The main goal of these experimental studies was determining the condition of the MoS_2_–treated grinding wheel active surface before and after the reciprocating internal cylindrical grinding process of rings made from INCONEL^®^ alloy 718. In the following sections, detailed information related to the general conditions in which the experimental studies were carried out, as well as selected results of the experiments along with their analyses, is given.

### 2.2. Characteristics of the Abrasive Tools and Workpiece

**Figure 2:**
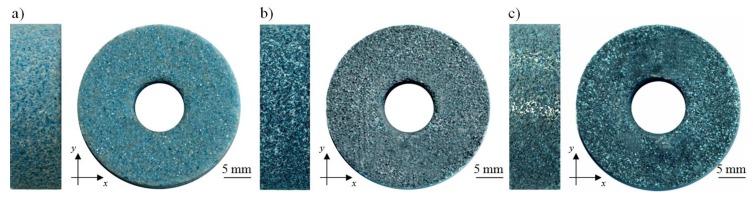
General view of 1-35×10×10×109A5X60L10VE01PI-50 grinding wheels used in the experimental studies: (**a**) M—non-impregnated reference wheel; (**b**) 1M40—MoS_2_-treated wheel; (**c**) 2M20—MoS_2_-treated wheel after the internal cylindrical grinding of INCONEL^®^ alloy 718.

Twenty small-sized grinding wheels with technical designation 1-35×10×10×109A5X60L10VE01PI-50 (Andre Abrasive Articles Sp. z o. o., Sp. k., Koło, Poland) were designated for experimental studies. Their general characteristics are given in Table 2, whereas a general view of the example wheels (M, 1M40, and 2M20) is presented in Figure 2. Eighteen grinding wheels were subjected to the impregnation process by MoS_2_, whereas two were used as a reference.

The impregnation process was modified. The authors propose a new method, described also by Wojtewicz [40], in which the introduction of an impregnating substance into the GWAS relies on dipping the grinding wheel in a prepared suspension of MoS_2_ (the internal phase). At the external phase, an organic dissolvent with a small quantity of lacquer can be used. The formation of a thin layer of impregnate which adheres to the grinding wheel grits is the final step of this method. In the described case, for the introduction of the impregnation substance into the structure of the GWAS, a weighed portion of solid grease (15–25 g of MoS_2_) was prepared. From the weighed portions, a suspension was prepared. The suspension, which consisted of 50 mm^2^ of a nitro thinner RS-1 and 25 mm^2^ of semi-matte nitrocellulose multicoat lacquer Solak NC 352520 (Sopur, Bydgoszcz, Poland), constituted the external phase (carrier), whose characteristics are given in Table 3.

Additionally, the suspension of nitro thinner and lacquer was prepared for the determination of residues of the external phase in the grinding wheel. All grinding wheels were impregnated in each of the suspensions. As an impregnation substance, the commercially produced MoS_2_ under the trade name Molykote^®^ microsize powder (Dow Corning Corp., Auburn, MI, USA) was used. Its characteristics are given in Table 4. 

Prepared in the way described above, the grinding wheels were used for the process of internal cylindrical grinding of the workpieces. In this case, as the workpieces, the rings (dimensions: *d_s_* = 50 mm, *d_w_* = 42 mm, *b_w_* = 20 mm) made of the advanced hard-to-cut material INCONEL^®^ alloy 718 were used. The chemical composition of INCONEL^®^ alloy 718 and selected properties are given in Table 5.

### 2.3. Conditions of the Grinding Process

**Figure 3:**
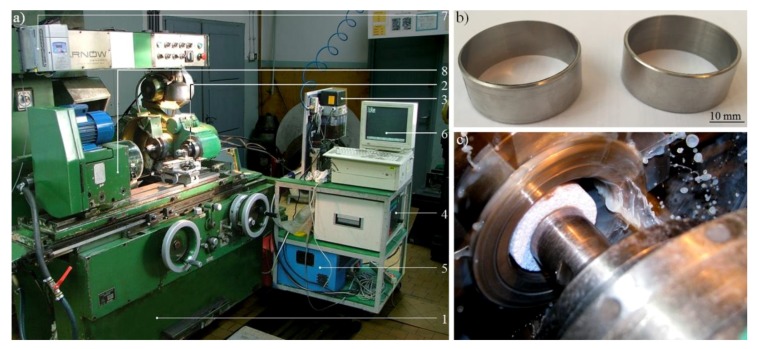
Experimental studies—set-up and workpiece: (**a**) general view of the experimental set-up used for realizing the internal cylindrical grinding process; (**b**) general view of the rings made of INCONEL^®^ alloy 718; (**c**) grinding wheel 1-35×10×10×109A5X60L10VE01PI-50 during machining of the sample. Note: In Figure 3a the main elements of the experimental set-up are marked: 1—universal grinding machine RUP 28P; 2—high-speed spindle EV-70/70-2WB; 3—dosage system of air–oil mixture IG 54-2; 4—frequency converter 21.60 (Sieb & Meyer AG, Lüneburg, Germany); 5—IK-V07 cooling unit; 6—computer with control software; 7—frequency converter SJ100 controlling the direct-current motor of workpiece spindle; 8—workpiece spindle.

The grinding process was carried out in the conditions collectively presented in Table 6, whereas the general view of the experimental set-up and workpieces is presented in Figure 3.

### 2.4. Contact and Non-Contact Measurements of the Grinding Wheels

The necessity of obtaining a reliable set of experimental results forced the use of a combination of many measuring techniques (stylus profilometry, optical profilometry, confocal laser scanning microscopy, and (environmental) scanning electron microscopy), represented by the list of observation measurement systems in Table 7.

Additionally, for processing and analysis of the GWAS images, the authors used Image-Pro^®^ Plus 5.1 (Media Cybernetics, Inc., Rockville, MD, USA) and STREAM Motion Desktop 1.8 (Olympus Corp., Shinjuku, Tokyo, Japan) software.

## 3. Results

### 3.1. Profilometry-Based Analysis of the GWAS Microgeometry

According to the data given in Table 7, the contact and non-contact measurements of GWAS microgeometry of all samples used in the experimental studies were carried out. In this case, the stylus profilometer Hommelwerke Hommel-Tester T8000 and optical profilometer Taylor Hobson TalySurf CLI2000 were used. Regardless of the instrument type, the surface texture measurements were performed using the same conditions. On each grinding wheel, ten evenly spaced areas with dimensions of 6 × 3 mm were selected. In these areas, the measurements were carried out.

**Figure 4:**
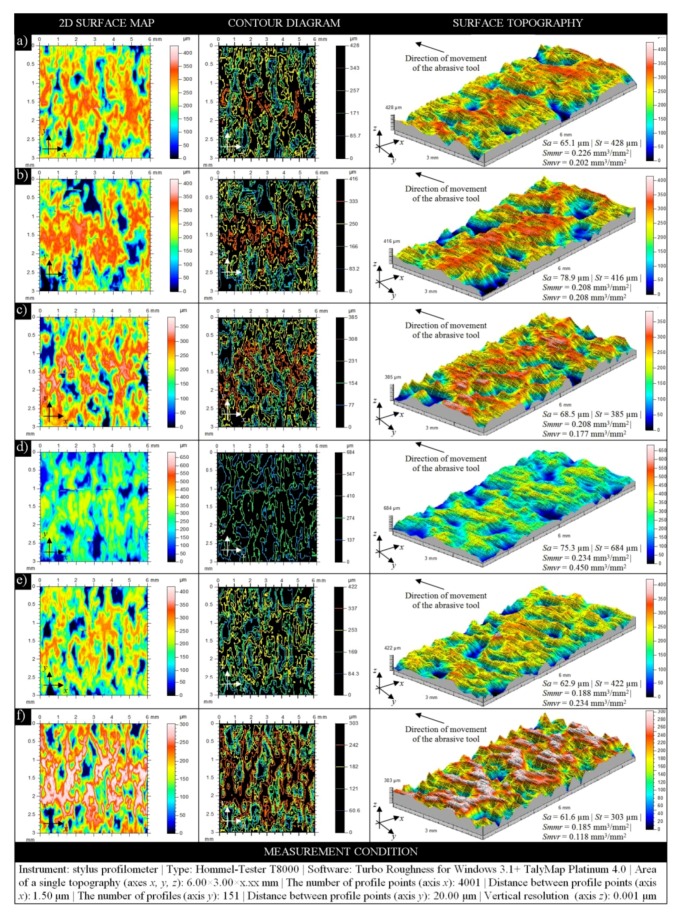
Collection of selected results obtained for the GWAS 1-35×10×10-9A5X60L10VE01PI-50 carried out using the stylus profilometer Hommelwerke Hommel-Tester T8000 for MoS_2_-treated samples—(**a**) 1M20, (**b**) 1M30, and (**c**) 1M40, as well as MoS_2_-treated samples after the internal cylindrical grinding of INCONEL^®^ alloy 718—(**d**) 2M20, (**e**) 2M30, and (**f**) 2M40.

A representative collection of results obtained through the use of stylus profilometer Hommelwerke Hommel-Tester T8000 is presented in Figure 4. Regardless of the surface conditions of each sample, the results presented include a two-dimensional (2D) surface map (in indexed colors), a contour diagram (color curves mode), and three-dimensional (3D) surface topography with a calculation of selected basic amplitude and volume parameters. Presented in this way, these results allow analyzing the characteristics of the surface texture of MoS_2_-treated grinding wheels before (Figure 4a–c) and after (Figure 4d–f) the internal cylindrical grinding process of INCONEL^®^ alloy 718. In both cases, the obtained parameter values had a decreasing tendency. The introduction of an impregnating substance with variable concentration into the structure of the abrasive tool resulted in a slight lowering of the height of surface irregularities in comparison to the non-treated tool.

A similar tendency in relation to the values of calculated parameters dominated also in the case of abrasive processing of this type of MoS_2_-treated tool. During such processing, numerous and often large-sized cloggings of the machined material occurred. Such a phenomenon is very unfavorable and has a major impact on the course and effects of the machining process (on the condition of the workpiece’s surface, as well as the condition of the GWAS and, thus, the abrasive tool life). Clogged areas generated on the GWAS caused an increase in the height of surface irregularities, which could be explained by the harsh machining conditions of the hard-to-cut material used in the experimental studies.

The TalyMap Platinum 4.0 software, used for visualization of the data presented in Figure 4, was also helpful in calculating selected parameters of the surface texture for MoS_2_-treated grinding wheels before (1M20–1M40) and after (2M20–2M40) the abrasive process. For the analysis of surface microgeometry of the above abrasive tools, the authors propose a set of parameters from two groups—amplitude (surface) *Sa, Sq, St, Sz,* according to ISO 25178-2:2012 standard [46] and EUR 15178 EN report [47], and roughness (profile) *Ra, Rp, Rq, Rz,* according to ISO 4287:1997 standard [48].

**Figure 5:**
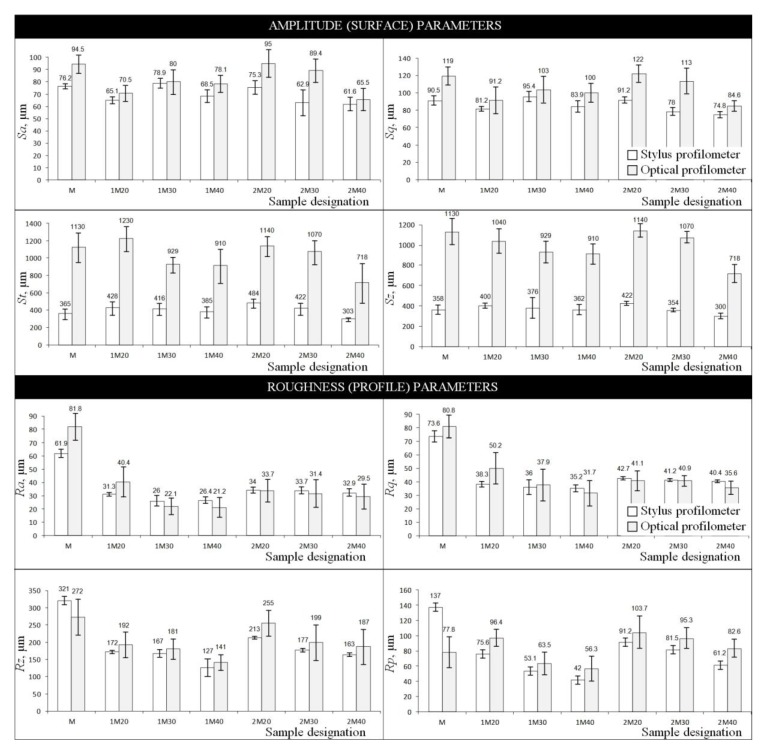
Values of selected (**a**) amplitude (surface) parameters, and (**b**) roughness (profile) parameters calculated on the basis of contact and non-contact measurements carried out using the stylus profilometer Hommelwerke Hommel-Tester T8000 (white bars) and optical profilometer Taylor Hobson TalySurf CLI2000 (gray bars) for samples used in the experimental studies.

These parameters are presented in Figure 5. The values obtained using a stylus instrument are presented in the form of white bars, whereas those obtained using an optical instrument are presented as gray bars. The reference value (M) represents data obtained for the non-treated grinding wheel, which was not involved in the abrasive process. In addition, standard deviation values were plotted on the data bars.

The analysis of parameters given in Figure 5 allows concluding that their values generally decreased depending on the concentration of impregnate substance introduced into the GWAS. A higher concentration of MoS_2_ in the given sample (M20—*C_i_* = 31.5%, M30—*C_i_* = 47.2%, M40—*C_i_* = 63%) resulted in relatively lower values of surface texture parameters, regardless of whether the sample took part in the abrasive process or not (the only exceptions were values of the *Sa* and *Sq* parameters calculated for samples 1M20–1M40). The difference between decreasing values (for samples 1M20–1M40 and 2M20–2M40) generally amounted to several to several hundred micrometers. Larger differences were noted for amplitude (surface) parameters, whereas lower differences existed for roughness (profile) parameters; the downward trend for the latter was much more visible. The differences between the abovementioned values in relation to the reference values (M) were on a similar level, i.e., from several to several hundred micrometers (the only exceptions were values of the *St* and *Sz* parameters).

In Figure 5, values of parameters obtained using the stylus profilometer Hommelwerke Hommel-Tester T8000 and optical profilometer Taylor Hobson TalySurf CLI2000 are given. It is interesting to analyze the differences resulting from the use of different measurement methods. Stylus profilometry was a reference method in this case, whereas the optical profilometry was used additionally. The difference between the two amplitude (surface) parameters *Sa* and *Sq* was relatively low and amounted to averages of ~30 μm (sample M) and ~35 μm (for samples 1M20–1M40 and 2M20–2M40), whereas, for other parameters from this group, *St* and *Sz*, the difference was higher and amounted to averages of ~400 μm to ~800 μm. For roughness (profile) parameters, no such high differences were noted as above. For the *Ra* parameter, the difference was relatively low and amounted to averages from ~5 μm to ~9 μm (samples 1M20–1M40) and from ~0.3 μm to ~3 μm (samples 2M20–2M40); the difference calculated between reference values was 30 μm (sample M). For the *Rq* parameter, calculated differences were at a similar level. In relation to other parameters (*Rz*, *Rp*), differences were higher and amounted to ~14 μm to ~42 μm (samples 1M20–1M40 and 2M20–2M40), as well as to ~10 μm to ~21 μm (samples 1M20–1M40 and 2M20–2M40), respectively. The differences between reference values in these two cases amounted to 49 μm (*Rz*) and 59.20 μm (*Rp*). The obtained results should be considered as reliable. The differences in the calculated parameters (varying from few to several hundred micrometers) in the case of the two different measurement methods correctly reflect the real characteristics of the GWAS, both in the pre-machining and post-machining conditions.

The most favorable results were obtained for samples which were characterized by the highest concentration of introduced impregnating substance (M40). Analyzing the amplitude (surface) parameters obtained for these samples, it can be stated that, by introducing the impregnating substance, the height of surface irregularities, in relation to the reference sample (M), was reduced by 7% to 10% (pre-machining condition) and by 16% to 19% (post-machining condition). For roughness (profile) parameters, the reduction of the height of surface irregularities was much higher and amounted to 30% to >40% (pre-machining condition) and to 40% to >50% (post-machining condition).

### 3.2. CLSM-Based Analysis of the GWAS Structure

**Figure 6:**
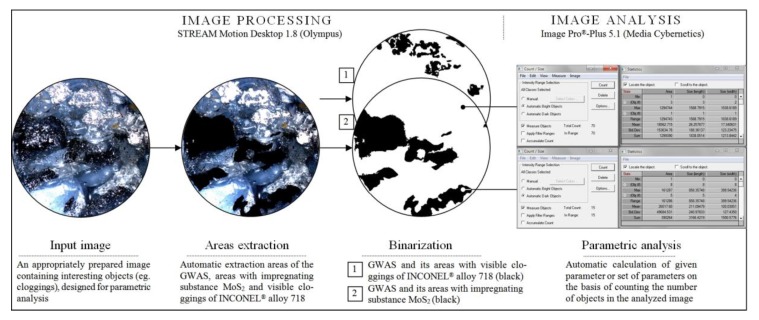
Procedure of image processing and analysis realized by Olympus STREAM Motion Desktop 1.8 and Media Cybernetics Image Pro^®^-Plus 5.1 software in relation to images of the 1-35×10×10×109 A5X60L10VE01PI-50 GWAS acquired using a three-dimensional (3D) laser microscope Olympus LEXT OLS4000.

The confocal laser scanning microscope (CLSM) LEXT OLS4000 (Olympus Corp., Shinjuku, Tokyo, Japan), supported by advanced image processing and analysis software, was used for studies aimed at determining the correctness of introduction of the impregnating substances into the GWAS structure, as well as the percentage share of MoS_2_-treated areas in relation to non-treated areas. Additionally, the percentage share of areas clogged by machined material in relation to non-treated areas was also determined. The above tasks were implemented on the basis of acquired real images of selected areas of the active surfaces of all abrasive tools used in the experimental studies. The active surface of each grinding wheel was analyzed in the 10 most interesting areas (e.g., covered by MoS_2_, containing clogging, microchips, and worn abrasive grains, etc.). Images acquired for a given GWAS were pre-processed using the Olympus STREAM Motion Desktop 1.8 software, and the full analysis was carried out using the Media Cybernetics Image Pro^®^-Plus 5.1 software. The processing procedure is graphically shown in Figure 6. 

**Figure 7:**
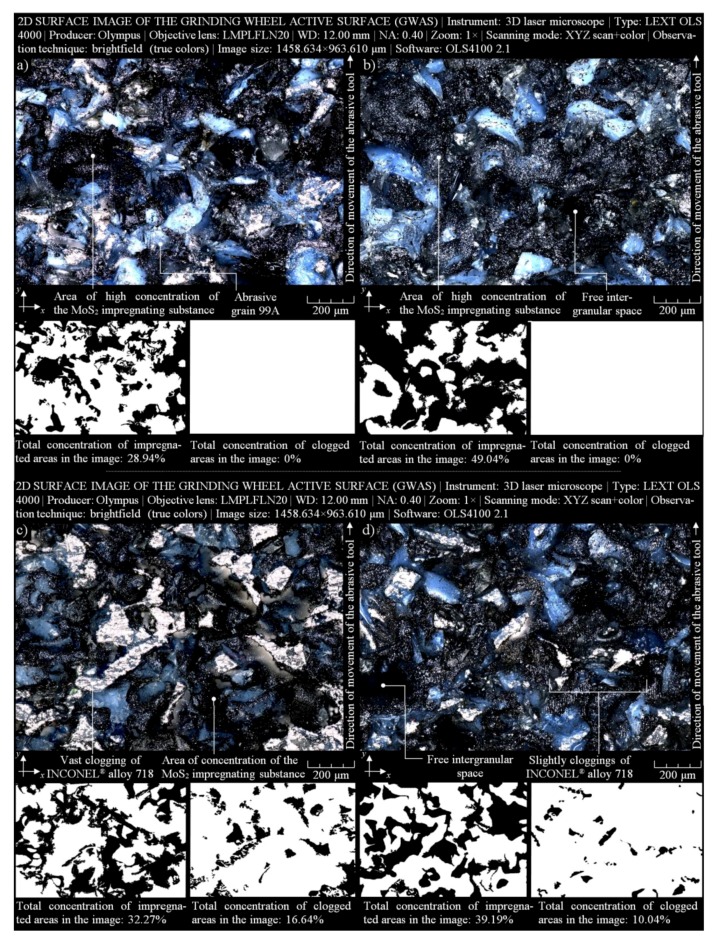
Collection of selected results obtained for the GWAS 1-35×10×10-9A5X60L10VE01PI-50 using a 3D laser microscope Olympus LEXT OLS4000, analyzed by Media Cybernetics Image Pro^®^-Plus 5.1 software for MoS_2_-treated samples—(**a**) 1M30 and (**b**) 1M40, and MoS_2_-treated samples after the internal cylindrical grinding of INCONEL^®^ alloy 718—(**c**) 2M20 and (**d**) 2M40.

The collection of selected results obtained using the above-described procedure is presented in Figure 7. Images acquired using the brightfield (true colors) technique, presenting 1458.63 × 963.61 μm fragments of the GWAS of MoS_2_-treated samples, are shown at the top (Figure 7a,b). The high quality of both images allowed precise analysis of selected elements of the GWAS, such as abrasive grains, free intergranular spaces, and areas with a high concentration of MoS_2_ impregnating substance (dark color). Corresponding to the above images, their variants after the binarization process were helpful with the calculation of the percentage share of MoS_2_-treated areas in relation to non-treated areas. In this case, the shares were 28.94% and 49.04% for samples 1M30 (Figure 7a) and 1M40 (Figure 7b), respectively. As those samples were in a pre-machining condition, no areas of the clogging of machined material were observed on the GWAS. 

Using the same microscopy technique and imaging parameters, another pair of GWAS images of MoS_2_-treated samples was acquired. The images are presented at the bottom of Figure 7. The GWAS in both cases was after the internal cylindrical grinding of INCONEL^®^ alloy 718. The characteristic remains of this process involved vast clogged areas of machined material (bright color). As previously described, the corresponding with image variants after the binarization process allowed calculating the percentage share of MoS_2_-treated areas in relation to non-treated areas and the percentage share of areas clogged by machined material in relation to non-treated areas. The obtained values were as follows: 32.27% and 39.19% (MoS_2_-treated areas), as well as 16.64% and 10.04% (clogged areas) for samples 2M20 (Figure 7c) and 2M40 (Figure 7d), respectively.

The size of analyzed GWAS areas, apart from giving their percentage share, was also characterized by a number of selected geometrical parameters, such as surface area (*An*), perimeter (*P*), length (*l*), width (*w*), minimal value of the Feret diameter (*F_min._*), and maximal value of the Feret diameter (*F_max._*). The values of those parameters calculated from acquired images of the GWAS (Figure 7a–d) are given in Table 8. For the first two fragments of GWAS of MoS_2_-treated samples (Figure 7a,b), differences in the determined geometric values (especially *An*) resulted from the MoS_2_ concentration percentage (for 1M30—*C_i_* = 47.2%, and for 1M40—*C_i_* = 63%). The value for 1M30 (*An* = 574,421.73 μm^2^) increased by 37.13% for 1M40 (*An* = 787,750.43 μm^2^), while the concentration of MoS_2_ increased by 33.47%. In the case of the subsequent two fragments of GWAS of MoS_2_-treated samples after the internal cylindrical grinding of INCONEL^®^ alloy 718 (Figure 7c,d), differences in the determined geometrical values (especially *An*) were also due to the percentage concentration of MoS_2_ and the size of vast clogged areas. The value for 1M30 (*An* = 530,242.60 μm^2^) increased by 45.93% for 1M40 (*An* = 773,795.65 μm^2^), at the same percentage of MoS_2_ concentration as above. The values of extracted clogged areas were *An* = 45,518.26 μm^2^ and *An* = 28,318.26 μm^2^, respectively. Therefore, this means that the introduction of the MoS_2_ impregnating substance effectively reduced the clogged areas by 37.78%.

### 3.3. SEM-Based Analysis of the GWAS Structure

**Figure 8:**
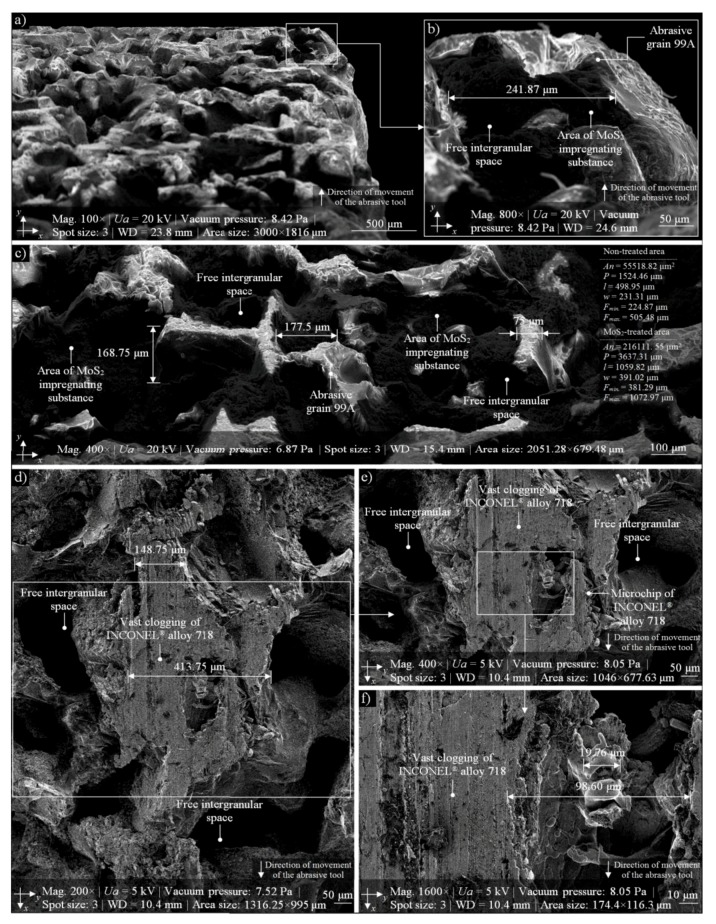
Collection of selected results obtained for the GWAS 1-35×10×10-9A5X60L10VE01PI-50 using the (environmental) scanning electron microscope FEI Quanta™ 250 for MoS_2_-treated samples: (**a**) SEM micrograph (area size: 3000 × 1816 μm, magnification: 100×) of GWAS fragment located on the right side of the grinding wheel (sample 1M40); (**b**) SEM micrograph (area size: 2051.28 × 679.48 μm, magnification: 400×) extracted from Figure 8a with clearly visible MoS_2_ impregnating substance; (**c**) SEM micrograph (area size: 2051.28 × 679.48 μm, magnification: 400×) of a vast panorama of another fragment of sample 1M40; (**d**) SEM micrograph (area size: 1316.25 × 995 μm, magnification: 200×) of the GWAS of sample 2M30 with a vast area of clogging of the machined material located in its center; (**e**) SEM micrograph (area size: 1046 × 677.63 μm, magnification: 400×) extracted from (**d**); (**f**) SEM micrograph (area size: 174.40 × 116.30 μm, magnification: 1600×) extracted from (**e**).

In many researches, SEM imaging is a complement or extension of the experiments. In order to observe the structures of the analyzed GWAS in high magnification, the authors decided to use this microscopic technique. In this part of the experimental studies, an advanced (environmental) scanning electron microscope FEI Quanta™ 250 was used. In Figure 8, selected results of SEM imaging obtained by means of the abovementioned microscope are shown. The SEM micrograph (area size: 3000 × 1816 μm, magnification: 100×) from Figure 8a presents a panoramic look at the GWAS fragment located on the right side of the analyzed 1-35×10×10-9A5X60L10VE01PI-50 grinding wheel (sample 1M40). The image perfectly reflects the characteristics of the surface, with clearly visible 99A abrasive grains and MoS_2_ impregnating substance. From this SEM micrograph, a fragment was extracted (area size: 405.76 × 329.48 μm, magnification: 800×) representing the MoS_2_ impregnating substance (dark color). In a slightly wider perspective, the structure of the GWAS from the previous Figure 8b is shown in Figure 8c. On this SEM micrograph (area size: 2051.28 × 679.48 μm, magnification: 400×), we can perfectly observe and analyze areas filled with MoS_2_ impregnating substance (dark color). On the right side of this figure, the selected geometrical parameters calculated with Media Cybernetics Image Pro^®^-Plus 5.1 software for MoS_2_-treated areas and non-treated areas (abrasive grains), as well as some selected dimensions of characteristic elements of the GWAS, are given. The SEM micrograph (area size: 1316.25 × 995 μm, magnification: 200×) from Figure 8d presents a fragment of the analyzed 1-35×10×10-9A5X60L10VE01PI-50 grinding wheel (sample 2M30) with a visible vast area of clogging of the machined material (INCONEL^®^ alloy 718). A more detailed analysis of this clogging can be traced on subsequent SEM micrographs (Figure 8e,f) at magnifications of 400× and 1600×, respectively.

## 4. Conclusions

On the basis of the results of experimental studies carried out on MoS_2_-treated GWAS before and after the internal cylindrical grinding process of INCONEL^®^ alloy 718, the following detailed conclusions can be drawn:

- Despite the passage of time, the impregnation of the abrasive tools seems to be a constantly developing industry of modern material engineering. The number of industrial applications in which impregnated abrasive tools are used is still large and systematically growing. Classic impregnating substances such as sulfur (Figure 1) were extended with new promising impregnates based on, e.g., graphene, hexagonal boron nitride, and molybdenum disulfide (Table 1). The last of these was used in experimental studies carried out by the authors, which are reported in detail in this work.
- The MoS_2_ impregnation process of 1-35×10×10-9A5X60L10VE01PI-50 grinding wheels (Figure 2) was realized using a new method (Section 2.2.) described by Wojtewicz [40]. This method allowed obtaining satisfactory results in the form of a correct and even introduction of the impregnating substance into the GWAS, which was confirmed based on images obtained by CLSM (Olympus LEXT OLS4000) and SEM (FEI Quanta™ 250) observation measurement systems (Figure 7 and Figure 8).
- Results obtained using contact and non-contact methods allowed stating that the values of parameters used during the GWAS microgeometry analysis (Section 3.1.) generally had a downward trend in relation to the reference values. This means that the introduction of MoS_2_ impregnating substance decreased the average values of the amplitude (surface) parameters (determined using the stylus method) by ~7%, while the average values of the roughness (profile) parameters decreased by ~54%. For MoS_2_-treated samples after the internal cylindrical grinding of INCONEL^®^ alloy 718, a decrease in the surface texture parameters in relation to the reference values was also observed. The average values of the amplitude (surface) parameters measured after finishing the process (determined using the stylus method) were lower by ~12%, while the average values of the roughness (profile) parameters were lower by ~45% (Figure 5). Upon omitting the errors occurring during the measurements, the obtained results should be considered significant for the experiments.
- GWAS microgeometry was measured using stylus and optical profilometry. The analysis of the results of measurements obtained with both methods (Figure 5) allowed concluding that there was a relatively high agreement between them (differences did not exceed 10–15%), which indicated the proper selection of the apparatus for the measurement tasks being performed.
- In addition to the observations carried out using CLSM (Section 3.2.) and SEM (Section 3.3.) confirming the correctness of introduction of the impregnating substance into the GWAS structure, a number of geometrical analyses were realized with Media Cybernetics Image Pro^®^-Plus 5.1 software. Parametrically, the percentage share of MoS_2_-treated areas in relation to the non-treated areas was described (Table 8), as well as the percentage share of areas clogged by machined material (Figure 7).

Positive results of the experimental studies presented in this work will encourage the authors to continue research works in this area. Future research directions will be related to issues including the improvement and modification of the impregnation method, the use of new impregnating substances, carrying out experiments on other materials from the hard-to-cut family, and the use of other types of measurement systems.

## Figures and Tables

**Table 1:** Substances used for the impregnating process of grinding wheels.

Impregnating Substance	References	
**Common Name**	**Patent**	**Paper**
Sulfur	Harmann [14], Jones [15], Jackson [16], Gallagher [17], Krueger et al. [18]	Sakuma and Tado [19], Younis and Alawi [20], Salmon [21], Nadolny et al. [22]
Graphite	Hunsberger and Tucker [23], Sioui and Cohen [24]	Shaji and Radhakrishnan [25], Irani et al. [26], Alberts et al. [27], Tsai and Jian [9]
Amorphous carbon	Sienicki et al. [28]	Nadolny et al. [22,29]
Graphene	-	Ravuri et al. [30], Paven et al. [31]
Silicone	Maeda et al. [32]	Kapłonek and Nadolny [33]
Epoxy resin	Rowse and Nelson [34], Ito [35]	-
Molybdenum disulfide	Serdyuk et al. [36], Bo et al. [37], Hashimoto and Iketani [38], Zhiqi et al. [39]	Wojtewicz [40]
Hexagonal boron nitride	Mathewson and Owens [41]	-
Bismuth alloy	Meyer [42]	-

**Table 2:** General characteristics of the grinding wheels used in the experimental studies.

**Technical Designation**	1-35×10×10×109A5X60L10VE01PI-50					
**Producer**	Andre Abrasive Articles Sp. z o. o., Sp. k. (Koło, Poland)					
**Grinding wheel type**	Flat grinding wheel					
**Dimensions**	External diameter *d_s_* = 35 mm, width *b_s_* = 10 mm, inner diameter *h_s_* = 10 mm					
**Abrasive grain type**	Microcrystalline sintered corundum SG™ (Norton, Worcester, MA, USA)					
**Abrasive grain fracture number (No.)**	60					
**Hardness class**	G					
**Structure No.**	10					
**Bond**	Vitrified (glass–crystalline)					
**Volume of grains (*V_g_*) **	42.0%					
**Volume of bond (*V_b_*)**	11.5%					
**Volume of pores (*V_p_*)**	46.5%					
**Experimental studies**	Designation	M	Pieces	2	Surface condition	Reference grinding wheel, non-impregnated
		1M20 ^1^		3		Impregnated by MoS_2_, after the dressing cut
		1M30 ^2^		3		
		1M40 ^3^		3		
		2M20 ^1^		3		Impregnated by MoS_2_, after the reciprocal peripheral internal cylindrical grinding of INCONEL^®^ alloy 718
		2M30 ^2^		3		
		2M40 ^3^		3		

Percentage concentration of the MoS_2_ impregnating substance: ^1^ M20—*C_i_* = 31.5%, ^2^ M30—*C_i_* = 47.2%, ^3^ M40—*C_i_* = 63%, according to Reference [40].

**Table 3:** Characteristics of semi-matte nitrocellulose multicoat lacquer Solak NC 352520.

Parameter	Value	Unit
Viscosity	4/25–35	mm/s
Density	0.90	g/cm^3^
Solid content	25	%
Color	colorless	-
Efficiency	10–12	m^2^

**Table 4:** Characteristics of the impregnation substance Molykote^®^ microsize powder.

Parameter	Value	Unit
Color ^1^	Black	-
Consistency	Free flowing powder	-
Purity (MoS_2_ content)	98	%
Particle size distribution (laser method: Malvern Mastersizer in 2-propanol)	90% <11.82 50% <5.22 10% <1.97	μm
Particle size (Fisher method)	0.65 to 0.80	μm
Service temperature range	−185 to +450	°C
Service temperature range (in hydrogen)	700	°C
Service temperature range (in vacuum)	Up to 1100	°C
Service temperature range (argon)	Up to 1300	°C
Theoretical density (solid state) ^2^	4.8	g/cm³
Bulk density (powder state) ^2^	0.5	g/cm³
Water/moisture content	0.05	%
Almen Wieland machine	>20,000	N
Press-fit test	0.06	μ

^1^ According to CTM 0176B; ^2^ according to ISO 2811-1:2016 [45].

**Table 5:** Chemical composition of INCONEL^®^ alloy 718 ^1^, as well as its selected physical, mechanical, and thermal properties.

Element	Concentration (%)	Physical Properties			
Ni + Co	50.00–55.00	Parameter		Value	Unit
Cr	17.00–21.00	Density		8.26	g/cm ^3^
Fe	Remaining	Melting range		1257–1342	°C
Nb + Ta	4.75–5.50	Modulus of	rigidity	77.2	kN/mm^2^
Mo	2.80–3.30		elasticity	204.9-122 ^2^	kN/mm^2^
Ti	0.65–1.15	**Mechanical Properties**			
Al	0.20–0.80	Elongation at break		-	<15%
Co	1.00 max.	Hardness (Brinell) HB		250–410	kg/mm^2^
C	0.08 max.	Yield strength		1030–640 ^4^	MPa
Mn	0.35 max.	Tensile strength		1280–780 ^4^	MPa
Si	0.35 max.	Elongation		12 ^4^	%
P	0.015 max.	**Thermal Properties**			
S	0.015 max.	Coefficient of thermal expansion		14.1–17.4 ^3^	μm/m
B	0.001–0.006 max.	Thermal conductivity		11.5–28.7 ^2^	W/m·°C
Cu	0.03–0.15 max.	Specific heat		460–658 ^2^	J/kg·°C

^1^ Used in the experimental studies; alloy was produced by Special Metals Corp. (New Hartford, NJ, USA) and distributed by Bibus Metals AG (Fehraltorf, Switzerland); ^2^ for temperatures in a range of 20–1200 °C; ^2,3^ for temperatures in a range of 20–900 °C; ^4^ for temperatures in a range of 20–800 °C.

**Table 6:** Conditions of the grinding process.

**Grinding Process**	Reciprocal Peripheral Internal Cylindrical Grinding
**Grinding machine**	Universal grinding machine: RUP 28P (Tarnów Mechanical Works SA, Tarnów, Poland), high-speed spindle: EV-70/70-2WB (Fisher Spindle Group AG, Herzogenbuchsee, Switzerland) with max. rotation 60,000 min^−1^; power of machine cutting = 5.2 kW
**Grinding wheel**	1-35×10×10×109A5X60L10VE01PI-50
**Grinding wheel dressing parameters**	Dresser: single grain diamond dresser with mass: *Qd* = 1.25 kt, Grinding wheel rotational speed while dressing: *n_sd_* = 10,000 min^−1^ Dressing allowance: *ad* = 0.0125 mm Axial table feed speed while dressing: *v_fd_* = 10 mm·s^−1^ Number of dressing passes: *i_d_* = 6
**Grinding parameters**	Grinding wheel peripheral speed: *v_s_* = 45 m·s^−1^ Axial table feed speed: *v_fa_* = 20 m·s^−1^ Working engagement (machining allowance): *a_e_* = 0.01 mm Total working engagement (machining allowance): *a_e tot_* = 0.75 mm Workpiece peripheral speed: *v_w_* = 0.56 m·s^−1^ Total grinding time *t_g tot_* = 900 s
**Grinding fluid**	5% water solution of Syntilo RHS (Castrol, Liverpool, Great Britain) oil given by flood method
**Workpieces**	Internal cylindrical surface of rings, made of INCONEL^®^ alloy 718 (dimensions: *d_s_* = 50 mm, *bw* = 42 mm, *b_w_* = 20 mm)

**Table 7:** Characteristics of observation measurement systems used in the experimental studies.

No.	System	Type	Producer	Configuration and Features
1.	Stylus profilometer	Hommel-Tester T8000	Hommelwerke GmbH (Villingen-Schwenningen, Germany)	Components: TKL100 pick-up with a diamond stylus tip (tip radius: *r* = 2.5 mm) + traverse unit Waveline™ 60 Basic (tracing length: *l* = 60 mm) + vertical displacement column Wavelift™ 400M (max. traverse: *l_max_* = 400 mm) + granite plate Wavesystem™ 780 + motorized positioning stage (Y-Positioner)
				Software: dedicated Turbo Roughness for Windows 3.1 + TalyMap Platinum 4.0 using Mountains Technology™ (Digital Surf, Besançon, France)
2.	Optical profilometer	TalySurf CLI2000	Taylor Hobson Ltd. (Leicester, Great Britain)	Components: LK-031 optical displacement sensor (wavelength: *λ* = 670 nm, power: *P_l_* = 0.95 mW, spot diameter: approximately 30 mm, resolution: 1 µm) + LK-2001 controller (Keyence, Osaka, Japan)
				Software: Talyscan CLI 2000 2.6 + Taly Map Silver 4.1 using Mountains Technonology™ (Digital Surf, Besançon, France)
3.	Confocal laser scanning microscope	LEXT OLS4000	Olympus Corp. (Shinjuku, Tokyo, Japan)	Components: motorized revolving nosepiece with a set of five objective lenses (BF Plan Semi Apochromat 5×, 10×, and LEXT-dedicated Plan Apochromat 20×, 50×, 100×) + motorized stage system ProScan III (Prior Scientific, Inc., Rockland, MA, USA)
				Software: dedicated OLS4100 2.1 (Olympus) + TalyMap Platinum 4.0 using Mountains Technology™ (Digital Surf, Besançon, France)
4.	(Enviromental) scanning electron microscope	Quanta™ 250	FEI Co., (Hillsboro, OR, USA)	Components: detectors: Everhadt-Thornley SED, large-field; low-vacuum SED (LFD), gaseous SED (GSED) (used in ESEM mode), BEI (solid-state (BSED)), 3-axis stage 50 × 50 × 50 mm, 8-port vacuum chamber, pressure: <6 × 10^−4^ Pa (HVM), <10 to 130 Pa (LVM) and <10 to 4000 Pa (ESEM vacuum), evacuation time: ≤150 s to high vacuum and ≤270 s to ESEM Features: magnification range: 14–1,000,000×, accelerating voltage: 0.2–30 kV, maximum horizontal FOV: 5 mm at WD = 10 mm, 8.8 mm at WD = 25 mm, resolution (using HVM): 0.8 nm at 30 kV (STEM), 1.0 nm at 30 kV (SE), 2.5 nm at 30 kV (BSE), 3.0 nm at 1 kV (SE); resolution (using LVM): 1.4 nm at 30 kV (SE), 2.5 nm at 30 kV (BSE), 3.0 nm at 3 kV (SE)
				Software: dedicated FEI software

**Table 8:** Selected geometrical parameters calculated with Media Cybernetics Image Pro^®^-Plus 5.1 software, acquired using a three-dimensional (3D) laser microscope Olympus LEXT OLS4000 image of the grinding wheel active surface (GWAS) from Figure 7.

**Sample**		**1M30**		**1M40**	
		**T**	**T + M**	**T**	**T + M**
**Parameter**	**Unit**	**Value**			
*An*	μm^2^	574,421.73	-	787,750.43	-
*P*	μm	9994.78		8469.56	
*l*	μm	1455.96		1537.46	
*w*	μm	999.91		1012.39	
*F_min._*	μm	893.04		885.05	
*F_max._*	μm	1546.90		1607.17	
**Sample**		**2M20**		**2M40**	
		**T**	**T + M**	**T**	**T + M**
**Parameter**	**Unit**	**Value**			
*An*	μm^2^	530,242.60	45,518.26	773,795.65	28,318.26
*P*	μm	4873.89	1866.68	3800.86	477.08
*l*	μm	1370.90	457.15	805.83	190.77
*w*	μm	898.59	277.36	549.89	86.58
*F_min._*	μm	882.77	274.22	531.68	79.00
*F_max._*	μm	1371.05	460.00	833.20	1463.00

T—MoS_2_-treated, T + M—MoS_2_-treated after the grinding process.

## Nomenclature

BEI	Backscattered electron imaging
BSE	Backscattered electron mode
CF	Cutting force
CL	Cooling liquid
CLSM	Confocal laser scanning microscopy
CTM	Corporate test method
ESEM	Environmental scanning electron microscope
FOV	Field of view
GSED	Gaseous secondary electron detector
GWAS	Grinding wheel active surface
GZ	Grinding zone
HVM	High-vacuum mode
ISO	International organization for standardization
LVM	Low-vacuum mode
SE	Secondary electron
SED	Secondary electron detector
STEM	Scanning transmission electron microscope
WD	Working distance
*a_d_*	Dressing allowance, mm
*a_e_*	Working engagement (machining allowance), mm
*a_e tot_*	Total working engagement (machining allowance), mm
*b_w_*	Width, mm
*d_s_*	External diameter, mm
*d_w_*	Inner diameter, mm
*i_d_*	Number of dressing passes
*l*	Length, μm
*n_sd_*	Grinding wheel rotational speed while dressing, min^−1^
*t_g tot_*	Total grinding time, s
*v_fa_*	Axial table feed speed while grinding, mm·s^−1^
*v_fd_*	Axial table feed speed while dressing, mm·s^−1^
*v_s_*	Grinding wheel peripheral speed, m·s^−1^
*v_w_*	Workpiece peripheral speed, m·s^−1^
*w*	Width, μm
*An*	Surface area, μm^2^
*C_i_*	Mass concentration of impregnate in the suspension, %
*F_min._*	Minimal value of the Feret diameter, μm
*F_max._*	Maximal value of the Feret diameter, μm
*P*	Perimeter, μm
*Q_d_*	Diamond dresser mass, kt
*Ra*	Arithmetic mean deviation of the roughness profile, μm
*Rp*	Maximum profile peak height, μm
*Rq*	Root-mean-square deviation of the roughness profile, μm
*Rz*	Average maximum height of the profile, μm
*Sa*	Arithmetic mean deviation of the surface, μm
*Smmr*	Mean material volume ratio, mm^3^/mm^2^
*Smvr*	Mean void volume ratio, mm^3^/mm^2^
*Sq*	Root-mean-square deviation of the surface, μm
*St*	Total height of the surface, μm
*Sz*	Maximum height of the surface, μm
*Ua*	Accelerating voltage, kV
*V_b_*	Volume of bond, %
*V_g_*	Volume of grains, %
*V_p_*	Volume of pores, %
